# More inclusive research is needed for an equitable response to dementia

**DOI:** 10.1016/j.lanepe.2022.100329

**Published:** 2022-02-22

**Authors:** Sarah Amele, Srinivasa Vittal Katikireddi

**Affiliations:** MRC/CSO Social and Public Health Sciences Unit, University of Glasgow, United Kingdom

Dementia affects an estimated 55 million individuals globally and is currently the seventh leading cause of death, although it will undoubtedly grow further over coming years.^1^ The 2020 Lancet Commission identified 12 modifiable risk factors that can be targeted to prevent or delay dementia, many of which are more common among socially disadvantaged groups.^2^ However, health research still too often under-represents people from minoritised ethnic groups and the most socioeconomically disadvantaged. In the UK this has meant studies predominately include White individuals from affluent backgrounds, with less known about the impact of ethnicity and other social factors on dementia.

The recent paper by Bothongo and colleagues published in *The Lancet Regional Heath - Europe* describes the association between ethnicity, area-level deprivation, and dementia.^3^ They carried out a nested matched case-control studying using primary care health data from four East London boroughs.^3^ By harnessing the power of electronic health records, they were able to ensure individuals from minoritised ethnic groups and people living in the most deprived areas were included.^3^ According to the authors, this is the first study to describe the risk of dementia among South Asians in the UK, and showed that this population subgroup have an increased risk compared to the White population living in the same area.^3^

They found that both ethnicity and area-level deprivation were independently associated with dementia. Individuals of Black and South Asian ethnicity had a higher risk of dementia (odds ratio [OR]: 1.43, 95% CI: 1.31–1.56 and 1.17 95% CI: 1.06–1.29, respectively).^3^ However, they did not find evidence to suggest that the known modifiable risk factors for dementia explained these differences, and therefore the authors suggest it is more important to target interventions at factors associated with ethnicity and deprivation than the modifiable risk factors.^3^ The authors highlight the potential importance of stress and traumatic life events, which are more common among certain ethnic groups and in deprived areas, for dementia.^4^ Experiences of daily and institutionalised racism faced by individuals from minority ethnic groups could potentially impact cognitive function, which could explain the increased risk of dementia among minority ethnic groups.^4^ Poverty can also increase stress and has been directly linked to dementia.^5^

There are some important limitations to consider when interpreting these results. Firstly, the study did not include data on education, which is known to be associated with dementia and may be a more important measure of socioeconomic position than area-level deprivation for cognition-related outcomes.^2^ The authors argue that education would be unlikely to account for ethnic differences, as the effect of education on dementia in the Lancet commission was estimated based on having no secondary education, and 99.8% of individuals in the UK have some secondary education;^6^ however, it is likely that differences in tertiary education may still matter.^7^ Secondly, while the proportion of missing ethnicity data was low in the study population (5.6%), there is a high proportion of misclassification of ethnicity in UK electronic health records.^8^ According to the Nuffield Foundation, the true extent of ethnic inequalities are under-estimated due to poor ethnicity coding.^8^ It found that patients from minority ethnic groups were more likely to have different codes used on different occasions, which suggests a higher proportion of inaccurate coding among minoritised ethnic groups.^8^ Thirdly, data on ethnic groups were aggregated to broader categories (White, South Asian, Black, Other, and Unknown) and they did not report more specific ethnic groups. More granular data would have been useful, as there are likely to be differences in health outcomes within these broad ethnic groups. Greater investment in data infrastructure and guidance for the collection of high quality of ethnicity data is required to allow the routine monitoring of ethnic inequalities in health for policy and health service planning purposes, as well as facilitating more robust research.

Bothongo and colleagues’ paper highlights the importance of improving diversity in health research. The under-representation of minority ethnic groups remains an important concern,^9^ especially given the rapidly growing number of minority ethnic people among the elderly across much of Europe. The use of electronic health records provides a valuable means of addressing some of the limitations of primary data collection. However, the lack of information on important social variables highlights the need to push further and ideally link data across sectors. Future research should aim to understand the mechanisms through which these inequalities occur, necessitating more focus on the social determinants of health.^10^ Additionally, methods for measuring experiences of racism and exploring how broader institutional and structural forces shape health remain under-developed in epidemiological research. Ultimately, more evidence to inform policies and interventions to reduce the negative impact of social inequality and structural racism on dementia is sorely needed.

## Declaration of interests

SVK was co-chair of Scottish Government's Expert Reference Group on ethnicity and COVID-19. Except for the funding acknowledged above, we declare no other conflicts of interest.

## References

[1] World Health Organisation. Dementia. 2021. https://www.who.int/news-room/fact-sheets/detail/dementia. Accessed January 17 2022.

[2] Livingston G., Huntley J., Sommerlad A. (2020). Dementia prevention, intervention, and care: 2020 report of the Lancet Commission. Lancet.

[3] Bothongo P., Jitlal M., Parry E. (2022). Dementia risk in a diverse population: a single-region nested case-control study in the East End of London. Lancet Reg Health Eur.

[4] Coogan P., Schon K., Li S., Cozier Y., Bethea T., Rosenberg L. (2020). Experiences of racism and subjective cognitive function in African American women. Alzheimers Dementia Diagn Assess Dis Monit.

[5] Resende E.P.F., Llibre Guerra J.J., Miller B.L. (2019). Health and socioeconomic inequities as contributors to brain health. JAMA Neurol.

[6] OECD. Educational attainment of 25-64 year-olds (2020). 2020. https://www.oecd-ilibrary.org/education/educational-attainment-of-25-64-year-olds-2020_5becebdb-en. Accessed January 18 2022.

[7] Brayne C., Ince P.G., Keage H.A. (2010). Education, the brain and dementia: neuroprotection or compensation?. Brain.

[8] Scobie S, Spencer J, Raleigh V. Ethnicity coding in English health service datasets. Nuffield Trust 2021.

[9] Osuafor C.N., Golubic R., Ray S. (2021). Ethnic inclusivity and preventative health research in addressing health inequalities and developing evidence base. EClinicalMedicine.

[10] Katikireddi S.V., Lal S., Carrol E.D. (2021). Unequal impact of the COVID-19 crisis on minority ethnic groups: a framework for understanding and addressing inequalities. J Epidemiol Commun Health.

